# Movement Disorders in Multiple Sclerosis: An Update

**DOI:** 10.5334/tohm.671

**Published:** 2022-05-04

**Authors:** Ritwik Ghosh, Dipayan Roy, Souvik Dubey, Shambaditya Das, Julián Benito-León

**Affiliations:** 1 Department of General Medicine, Burdwan Medical College & Hospital, Burdwan, West Bengal, India; 2 Department of Biochemistry, All India Institute of Medical Sciences (AIIMS), Jodhpur, Rajasthan, India; 3 Indian Institute of Technology (IIT), Madras, Tamil Nadu, India; 4 School of Humanities, Indira Gandhi National Open University, New Delhi, India; 5 Department of Neuromedicine, Bangur Institute of Neurosciences, IPGME&R, Kolkata, West Bengal, India; 6 Department of Neurology, University Hospital “12 de Octubre”, Madrid, Spain; 7 Centro de Investigación Biomédicaen Red sobre Enfermedades Neurodegenerativas (CIBERNED), Madrid, Spain; 8 Department of Medicine, Complutense University, Madrid, Spain

**Keywords:** ataxia, dystonia, movement disorders, multiple sclerosis, tremor, restless leg syndrome, chorea/ballism, parkinsonism, tics, myoclonus

## Abstract

**Background:**

Multiple sclerosis (MS), a subset of chronic primary inflammatory demyelinating disorders of the central nervous system, is closely associated with various movement disorders. These disorders may be due to MS pathophysiology or be coincidental. This review describes the full spectrum of movement disorders in MS with their possible mechanistic pathways and therapeutic modalities.

**Methods:**

The authors conducted a narrative literature review by searching for ‘multiple sclerosis’ and the specific movement disorder on PubMed until October 2021. Relevant articles were screened, selected, and included in the review according to groups of movement disorders.

**Results:**

The most prevalent movement disorders described in MS include restless leg syndrome, tremor, ataxia, parkinsonism, paroxysmal dyskinesias, chorea and ballism, facial myokymia, including hemifacial spasm and spastic paretic hemifacial contracture, tics, and tourettism. The anatomical basis of some of these disorders is poorly understood; however, the link between them and MS is supported by clinical and neuroimaging evidence. Treatment options are disorder-specific and often multidisciplinary, including pharmacological, surgical, and physical therapies.

**Discussion:**

Movements disorders in MS involve multiple pathophysiological processes and anatomical pathways. Since these disorders can be the presenting symptoms, they may aid in early diagnosis and managing the patient, including monitoring disease progression. Treatment of these disorders is a challenge. Further work needs to be done to understand the prevalence and the pathophysiological mechanisms responsible for movement disorders in MS.

## Introduction

Resources appraising movement disorders in multiple sclerosis (MS) differ in clinical characteristics and accurate prevalence data [1,2,3]. This variation could stem from the retrospective nature of the studies, small sample size, and consideration of coexisting non-MS-related movement disorders, among others. Neuroinflammation and neurodegeneration are the two processes that pathophysiologically define MS [2]. Although movement disorders are common in MS, their accurate pathobiological basis remains elusive [2]. The interrelation between these two might be either causal or coincidental [4]. Some movement disorders present with symptoms highly suggestive of MS, which should prompt immediate diagnosis and treatment. The most prevalent movement disorders in MS include restless leg syndrome (RLS), tremor, ataxia, parkinsonism, paroxysmal dyskinesias, dystonia, chorea and ballism, facial myokymia, including hemifacial spasm and spastic paretic hemifacial contracture, tics, and tourettism [1,2,3,4,5,6].

Our aim with this review is: 1) to describe the entire spectrum of movement disorders associated with MS; 2) to discuss possible pathophysiological mechanisms responsible for movement disorders in MS and; 3) to discuss the therapeutic modalities of the various movement disorders in MS.

## Methods

We conducted a narrative literature review by searching for the keywords ‘multiple sclerosis’ and the specific movement disorder on PubMed until October 2021. The search terms are outlined in Supplementary ***Table 1***. Relevant articles were screened, selected, and included in the review according to groups of movement disorders.

**Table 1:** Clinical presentation and anatomical correlates of ataxia in multiple sclerosis.


PRESENTATION	LOCALIZED LESION

Gait ataxia; truncal ataxia; titubation	Midline of the cerebellum, including the vermis; cerebellar peduncle.

Gait ataxia; nystagmus; balance problems	Posterior cerebellum, including the flocculonodular lobe; cerebellar peduncle.

Limb ataxia	Cerebellar hemisphere; cerebello-rubro-thalamocortical tract.

Sensory ataxia	Vestibular system; central or peripheral sensory tracts.

Ataxic tremor	Cerebello-rubro-thalamocortical tract

Ataxic hemiparesis	Internal capsule

Paroxysmal dysarthria	Midbrain; dorsolateral pons

Paroxysmal limb hemiataxia	Upper pons; ventral central trigeminal tract; brachium conjunctivum; lateral spinothalamic tract


We undertook a descriptive analysis, where the movement disorders were separated into groups and discussed according to their prevalence, causal relation with MS, pathophysiology, and treatment modalities. After searching databases and initial screening titles and abstracts, two separate reviewers (R.G. and D.R.) extracted the relevant papers to screen full-text manuscripts. We omitted duplicate publications during this process. Search results were further augmented by bibliographic searches and studies familiar to the authors. The included studies were case reports, case series, literature reviews including systematic reviews and meta-analysis, population-based studies, and clinical trials related to any movement disorder associated with MS. Studies were excluded if they did not include human subjects. We extracted the data from the included publications.

## Results

### 1) Restless legs syndrome

There is a significant association between MS and RLS, especially in cases with severe sensory and motor disabilities [7,8,9,10,11,12,13,14,15,16]. RLS prevalence rates in patients with MS are significantly higher than in the general population, especially in women [7,8,9,10,11,12]. A recent meta-analysis reported a prevalence of RLS of 27.5% (13.2–65.1) in patients with MS [17].

RLS negatively impacts sleep quality and causes excessive daytime sleepiness [7,8,9,10,11,12,13,14,15,16,18]. Moreover, this resultant sleep impairment may be one of the causes of the cognitive decline associated with MS [9]. Therefore, a search for RLS should be done in patients with MS having insomnia.

The pathophysiologic link between MS and RLS is yet to be established, and several theories have been proposed [15,16,19]. The presence of cervical cord lesions is more common in patients with MS having RLS symptoms than in those who do not [20,21]. MS-related inflammatory damage may also induce secondary forms of RLS [11]. Iron deficiency anemia is a known risk factor for RLS with or without MS [20]. RLS is particularly found in premenopausal MS women because they have a higher likelihood of worse iron stores due to menstrual loss [22]. Ferritin level, when lower than 50 μg/L, should be considered a candidate for iron replacement in patients with RLS with or without MS [23].

Dopamine agonists, i.e., pramipexole, ropinirole, transdermal rotigotine, effectively manage RLS [24]. Clonazepam, gabapentin, and levodopa/carbidopa are the other options [25]. Therapy-related augmentation of RLS symptoms is an important clinical problem reported in up to three-fourths of patients treated with levodopa and, to a lesser extent, with dopamine agonists [26]. Co-activation of functionally different dopamine receptor subtypes or interactions with other receptors may have a role to play in augmentation [27]. Therefore, newer treatment options should consider these dynamic changes in the dopaminergic system. A recent clinical trial has provided preliminary evidence that a 16-week physical activity can effectively reduce RLS severity and improve sleep outcomes in MS patients [9].

### 2) Tremor

Tremor was included in the original triad of MS symptoms postulated by Charcot, i.e., tremor, scanning speech, and nystagmus [28,29]. The estimated prevalence of tremor is between 25% and 58% of the patients with MS, with 3–15% having severe MS-related tremors [28]. Patients with MS who have tremor of any severity retire early or become unemployed because of disability [28,29]. Typically a combination of postural and intention tremors may be observed in patients with MS [28,29]. Tremor in MS is often bilateral and involves the upper limbs more than the lower limbs, but can affect the head, neck, and even vocal cords [28,29,30]. The pathophysiological basis of tremor in MS is elusive, as it seldom occurs in isolation [28,29,30]. Because of the preponderance of mixed (postural and intentional) tremors and scarcity of rest tremor syndromes in MS, it likely originates from and is mediated by cerebellar connections [28,29,30]. The intensity of MS tremors can be reduced by cooling of extremities, likely due to a reduction in muscle excitability and neuronal conduction, causing reduced input to the cerebellar circuitry [28,29,30,31]. The severity of upper limb tremors is strongly associated with high degrees of ataxia, dysmetria, and dysdiadochokinesia, which may be due to an aberrant cerebellar-thalamocortical network [5,28,29,30,32]. Recently, this involvement has been supported by imaging studies. Increased lesion load and cerebellar and thalamic atrophy were observed on the ipsilateral side of the tremor than on the side without tremor in MS patients [33,34]. Other studies have found significant associations between tremor amplitude and increased contralateral pontine lesion load highlighting the role of pontine components in MS-tremor [35].

Holmes’ tremor (also termed “rubral” or “midbrain”) [5,36,37], a combination of kinetic, postural, and resting tremor, predominantly involving the proximal limbs, is uncommon in MS [38]. It appears due to cerebellothalamic and nigrostriatal pathway dysfunction, and it is partially responsive to levodopa [37].

**Figure 1:**
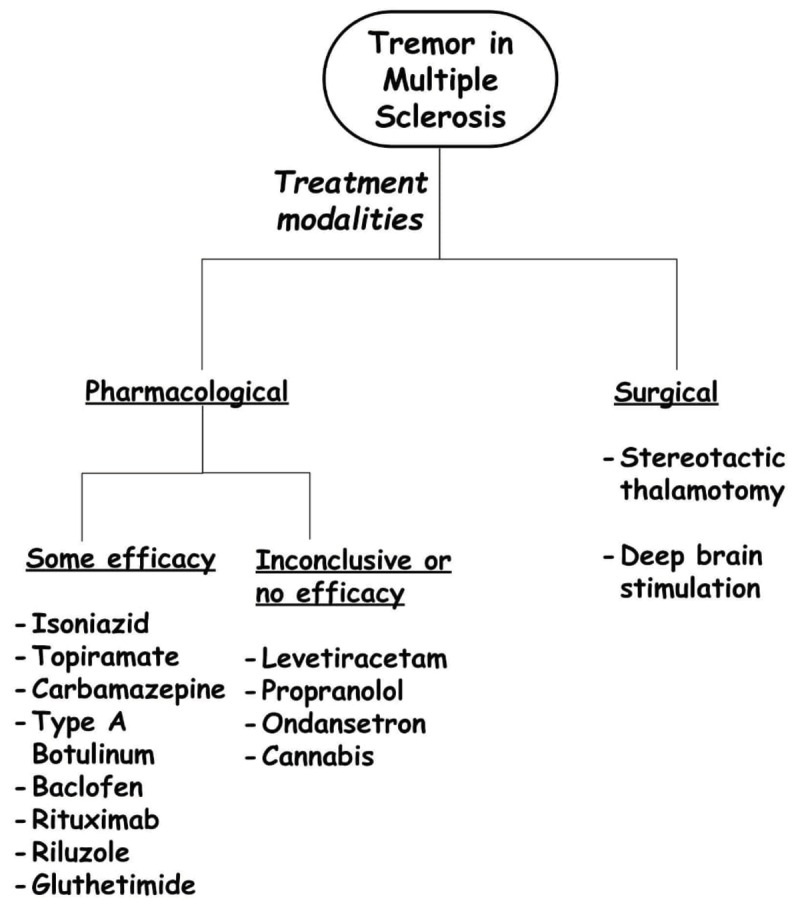
Treatment modalities in multiple sclerosis-associated tremors.

***Figure 1*** summarizes the treatment modalities for the tremor in MS. Isoniazid, one of the first-line anti-tubercular agents, has shown promising results in a few randomized controlled trials in MS-related tremors (***Figure 1***) [39,40,41,42,43,44,45]. However, several adverse effects have limited its use [39,40,41,42]. Results with cannabis/cannabinoids have not been remarkably successful [46,47,48]. 4-aminopyridine positively impacts MS-related tremors as it presumably improves the excitability of the cerebellar Purkinje neurons [49]. Anecdotal reports claim that topiramate may be helpful at low doses to control MS-tremor and enhance functionality [50]. Type-A botulinum toxin was also found to be effective in the treatment of MS-related tremors [51,52,53]. Other medications such as levetiracetam, ondansetron, primidone, propranolol, baclofen pump, and carbamazepine have shown conflicting results [51,52,53,54,54,55,56,57,58,59,60,61].

For the last 3–4 decades, surgical treatment for relieving tremor in MS have been tried with mixed success rates [30,55,62,63,64,64,65,66,67,68,69,70,71,72,73,74,75,76,77]. Surgical options include deep brain stimulation (DBS) and stereotactic thalamotomy [63,64,65,66,67,68,69,70,71,72,73,74,75,76]. As most studies are small, retrospective, and observational, they are insufficient to provide data on the efficacy of these interventions on post-intervention functional status, post-intervention residual disability, and long-term follow-up status of adverse events [63,64,65,66,67,68,69,70,71,72,73,74,75,76]. It is difficult to demonstrate an improvement in health-related quality of life with such interventions as it is difficult to differentiate between tremor-related and MS-related disabilities [78]. Overall, thalamic stimulation with DBS and thalamotomy had comparable effects in controlling tremors in MS [79]. Permanent reduction in MS-related tremors following DBS can also be attributed to the natural progression of the disease, where limb weakness prevents the expression of the tremor [80,81,82,83,84,85]. Recently, MRI-guided focused ultrasound thalamotomy was successfully used to treat a medically refractory tremor in a 28-year-old female [81]. The procedure is minimally invasive and has yielded positive results in patients with essential tremor [86]. However, in the reported case [81], the patient developed dysarthria that took nearly 12 months to resolve [81]. Imaging-guided thalamotomy, although proven to help manage tremors in MS, may be associated with neurological side effects, which may require a longer time to resolve. However, direct evidence of “off-target” damage in neuroaxis was not evident in earlier cases, demanding more rationale and further pragmatism in patient selection [81]. For treatment-resistant tremors in MS such as Holmes’ tremor, simultaneous DBS involving ventralis intermedius with globus pallidus internus pallidotomy may be efficacious due to the synergistic effect [87].

### 3) Ataxia

In isolation or associated with other neurological deficits, ataxia is a common manifestation (almost 80% of patients) in demyelinating disorders and significantly impacts health-related quality of life [25,88]. It is caused by dysfunction of the cerebellar connections with the cerebral cortex, brainstem, thalamus, and spinal cord [25,88].

It can be difficult to directly correlate clinical manifestations of cerebellopathies with cerebellar anatomy [89,90]. Midline cerebellar lesions result in gait and truncal ataxia and titubation, while paravermian area involvement affects speech. Posterior cerebellar lesion or flocculonodular lobe involvement is known to cause vertigo, ataxia, and eye movement abnormalities [89,90,91]. Limb ataxia is associated with ipsilateral cerebellar hemispheric lesions [89,90]. The lesions associated with these tremors may also involve the cerebellum-rubro-thalamocortical tract beyond the cerebellar cortex (the long-loop pathway) [89,90]. Sensory integration is another significant component of the coordination of voluntary movements. Hence, demyelinating lesions involving the central or peripheral sensory tracts and the vestibular system can cause sensory ataxia [89,90,92].

In a recent retrospective study involving 123 patients with movement disorders and demyelinating diseases, ataxia was the most common movement disorder, followed by an isolated tremor [6]. In a study involving five MS patients who developed limb ataxia/intention tremor, a contralateral cortical MRI lesion became visible on an average of 22.3 months before the development of limb ataxia [93]. Lesions involving the contralateral thalamus and internal capsule have also been reported in MS in the form of ataxic hemiparesis, almost indistinguishable from a vascular syndrome [94]. Episodic ataxia may also be present in demyelinating diseases [95]. A recent case report revealed a coincidental occurrence of type 2 episodic ataxia and MS in a patient with previously unidentified heterozygous mutation in the gene coding for the voltage-gated calcium channel subunit alpha 1A [96]. These cases highlight the various anatomical regions that can be affected in demyelinating lesions, resulting in ataxia and the long-term disability of ataxia in patients with MS.

Ataxia in MS is often associated with paroxysmal dysarthria (PDA) [97]. An episode of PDA is usually accompanied by slurring of speech and gait ataxia that may last seconds and recur throughout the day [97]. Sensory symptoms, such as numbness, burning sensations, and paresthesia of the face, tongue, or limbs, may follow and can be provoked by physical or emotional stress [95,97,98,99]. PDA can be attributed mainly to midbrain lesions at or below the level of the red nucleus but has also been described in dorsolateral pons and cerebellar lesions [95,99,100,101,102,103,104,105]. More than 60 cases of PDA in MS have been documented to date [99,103,105,106,107,108]. Furthermore, paroxysmal limb hemiataxia with crossed facial paresthesia may also occur in MS, albeit rarely [109,110]. The causative lesion is localized in the upper part of the pons, affecting the ventral central trigeminal tract, brachium conjunctivum, and lateral spinothalamic tract [110].

The treatment for ataxia is symptomatic and often multidisciplinary. The treatment options range from pharmacologic to physical, occupational, and speech therapy and rehabilitation. Multiple trials have been carried out with varied results [67,88,111,112,113], but ultimately failed to show conclusive evidence of a singular effective therapy. A systematic study comprised of 10 randomized controlled trials investigated a wide range of therapeutics, including baclofen, pyridoxine, isoniazid, cannabis, thalamotomy, DBS, physiotherapy, and neurorehabilitation in MS, none of which proved to be effective against ataxia in the long-term [88]. However, the assessment of outcomes in these studies was possibly affected by the different methods used to measure tremors and the small sample size [88]. Thalamic DBS and thalamotomy have shown initial promise in medication-resistant tremors in MS, but on follow-up, not only did the treatment group suffered from other adverse effects such as dysarthria and ataxia, but disability scores did not improve [67].

Even though physiotherapy has been shown to improve function in ataxia modestly, its long-term benefits in MS patients are unclear. Two different trials revealed an improved Expanded Disability Status Scale score and Rivermead Mobility index in patients with MS randomized to physiotherapy [111,113]. Another study on 42 randomized patients initially showed improvement in Rivermead Mobility Index for home and outpatient therapy groups compared to no therapy. However, after two months of follow-up, mobility regressed to pretreatment levels [112]. Balance-based torso-weighting has been beneficial in standing stability, cadence, gait velocity, and percentage of the gait cycle in single-limb support in MS patients [114,115,116] and in cerebellar ataxia patients [117]. Ali et al [118]. added core stability exercises and task-oriented training to traditional balance training in 45 ataxic relapsing-remitting patients with MS and concluded that this technique might improve stability.

Similarly, in a group of 42 patients with MS, task-oriented training and lumbar stabilization improved the success of balance rehabilitation [119], showing significant improvement in composite balance scores and the International Cooperative Ataxia Rating Scale. Finally, a targeted ballet program aimed at mitigating MS-associated ataxia and improving balance in women showed significant clinical improvement, as observed by the International Cooperative Ataxia Rating Scale, the Mini-Balance Evaluations Systems Test, the smoothness of movement on both sides in a five-meter walk, and balance in a step-to-stand task before and after the intervention [120]. All these studies point towards the promising benefits of physiotherapy in MS-related ataxia.

Pharmacologic treatment for cerebellar ataxia also remains challenging. Benzodiazepines and barbiturates (e.g., clonazepam, primidone), although effective in improving tremors, can cause worsening of balance and coordination in the long term [121]. Episodic ataxia can be effectively treated by acetazolamide and calcium-channel blockers [122]. Topiramate has shown significant functional improvement in a sustained, dose-dependent manner [50]. Levetiracetam has also been shown to significantly improve tremor and ataxia in a small pilot study of 14 patients with MS [56]. Standard antiepileptics, such as carbamazepine [99,100], levetiracetam [105], lacosamide [102], phenytoin [110], and acetazolamide [108] have been effective in PDA related to episodic events in MS. Goodwin and Carpenter [105] reported PDA in a 37-year-old woman approximately three months after a multifocal MS relapse. The lesions were in the posterior midbrain and the right posterior internal capsule [105]. Levetiracetam (500 mg twice daily) was administered, which reduced her attack frequency [105]. The attacks completely stopped when the dose was increased to 750 mg [105]. Similarly, a 49-year-old MS patient who developed PDA due to a midbrain lesion responded well to carbamazepine [101]. In another case [103], the PDA attacks resolved after fingolimod treatment. Fingolimod may have added benefits in MS patients with ataxia. However, immunosuppressive side effects must be weighed against potential benefit [123].

A summary of ataxia in MS and possible treatment options is provided in ***Tables 1*** and ***2***.

**Table 2:** Treatment modalities for the ataxia of multiple sclerosis patients.


TREATMENT MODALITY	OPTIONS

Physical	Balance-based torso weighting, task-oriented, and core-stability exercises

Pharmacologic	Carbamazepine, levetiracetam, phenytoin, acetazolamide, lacosamide, fingolimod^#^

Surgical*	Thalamic deep brain stimulation, thalamotomy


* Adverse effects such as dysarthria and ataxia; disability scores not improved. ^#^ To be used with caution as it has significant immunosuppressive effects.

### 4) Paroxysmal Dyskinesia and Other Paroxysmal Disorders

Intermittent hyperkinetic movement episodes with intact consciousness characterize paroxysmal dyskinesias. These include self-limiting episodes of dystonia, chorea, athetosis, or a combination of all of these. Paroxysmal dyskinesia can be primary (hereditary) or secondary (due to other causes). Secondary paroxysmal dyskinesia is most commonly observed in MS, either during the course or as the presenting symptom [124]. The attacks are of stereotypical pattern, brief (seconds to minutes), non-sustained, gradually increase in frequency over time, and can occur as much as 100 times a day [125,126,127,128,129]. Electroencephalogram is normal.

Paroxysmal dystonia consists of abrupt onset, involuntary muscle contractions causing stereotyped posturing, or repetitive and patterned twisted movements [130,131]. It can affect the face, arm, and leg and be precipitated by hyperventilation, tactile stimulation, voluntary movement, or emotional stressors [3]. Patients usually have unilateral upper limb flexion and lower limb extension episodes, potentially spreading to the neck or face [125,126,132]. An unpleasant sensory aura (ipsilateral or contralateral) can precede the episodes [124,133,134]. The pathophysiology has been attributed to axonal inflammation, axonal hypersensitivity, potassium channel alteration, decreased ionized calcium, and demyelination of the afferent inhibitory neuroanatomic pathways [130,135,136]. Demyelinating lesions lead to an ephaptic activation of secondary axons, especially where the motor fibers run closely together [109,134]. In the reported cases, lesions are detected in the midbrain, cerebral peduncle, thalamus, basal ganglia, contralateral posterior limb of the internal capsule, brainstem, and cervical spinal cord [125,131,135,136,137,138,139,140,141,142,143,144,145,146,147,148,149]. In a recent retrospective voxel-wise symptom mapping analysis of 25 patients with MS, paroxysmal dyskinesia was causally associated with basal ganglia lesions adjacent to the thalamus, the internal capsule, and the periventricular occipital area of the posterior thalamic radiations [150]. Thus, lesions in various regions may contribute to paroxysmal dystonia in MS. Generally, a normal electroencephalogram can exclude the possibility of the misdiagnosis of a focal onset seizure [124,135,149,151].

Tonic spasms are sudden, involuntary movements usually lasting seconds, manifesting with abnormal posturing of limbs or part of a limb and precipitated by voluntary movements, emotional stress, and specific sensory stimulation. Paroxysmal tonic spasm is rarely observed in demyelinating disorders like MS and neuromyelitis optica spectrum disorder [152]. Abnormal sensory integration in the thalamus, which may also stem from the ephaptic activation of neurons in the spinal cord, and related dopamine level fluctuations in basal ganglia are thought to be the basic underpinning mechanisms [152].

Focal dystonia cases that have been reported in MS include oromandibular [127,128], writer’s cramp [153], and pharyngeal forms [133]. Another type of focal dystonia, infrequently reported in MS, is cervical dystonia [154,155,156,157,158,159,160,161], which usually appears a few years after the onset of MS [3,147,162,163]. The association between cervical dystonia and MS was thought to be coincidental [148]. However, a causal association has also been implicated [146,158,159,160], in the form of high cervical spinal lesions on MRI, lesions in the left posterior putamen, and the patient’s response to corticotropin. Indeed, lesions in the cervical spinal cord can cause interruptions of afferent fibers responsible for the proprioception of head posture [164]. The treatment for cervical dystonia in MS varies depending on whether the attacks are related or not to an MS relapse [3,159,165].

A few MS-associated choreoathetosis cases have been reported [166,167,168,169,170,171,172,173]. In these cases, the lesions have been found in varied areas, such as the basal ganglia circuitry and mesencephalon [166,167,168,172,174], thalamo-striatal network [166,167], posterior part of the internal capsule [167], medial longitudinal fasciculi [169], and cervical cord [173].

Regarding treatment, paroxysmal dyskinesias in MS may be self-limiting and without the need for any therapeutic intervention. Classically, pulse steroids, alone or in combination with symptomatic treatments, are the treatment of choice [126,132,134,136,161]. Symptomatic treatment is necessary when movements persist despite immunosuppressive treatments [97]. Carbamazepine is one of the best options [130,147,151]. Other useful drugs are acetazolamide [132,175], clonazepam [175], levetiracetam [175], valproate [134], and oxcarbazepine [136]. For cervical dystonia, corticosteroids are the first line, whether there is an MS-relapse manifestation [3,163]. For gradual-onset attacks unrelated to MS exacerbation or persistent symptoms despite high-dose corticosteroids, botulinum toxin is the optimal treatment [159].

For MS-related choreoathetosis, therapeutic evidence is not well documented. However, corticosteroids, antiepileptics such as carbamazepine, oxcarbazepine, phenytoin, valproate, lacosamide, and neuroleptics such as haloperidol, risperidone, and olanzapine are among the options [97,121,176].

### 5) Myoclonus

The “Guillain Mollaret” triangle (GMT) (or myoclonic triangle) is formed by the red nucleus and inferior olive ipsilaterally connected to the contralateral cerebellar dentate nucleus. The red nucleus in the midbrain connects with the ipsilateral inferior olive in the medulla through the central tegmentum tract, traversing through the pons. This triangle includes almost the whole of the brain stem, which is packed with white matter tracts, and thus vulnerable to be affected by a demyelinating disease like MS. This brainstem and deep cerebellar nucleus connection modulate the spinal cord motor activities, thereby heralding varied neurological manifestations once acted. Only a handful of cases of myoclonus have been reported in MS [177,178,179,180,181,182,183,184,185,186,187]. The palatal type is the most commonly encountered [147], usually associated with nystagmus. Palatal myoclonus is a form of segmental myoclonus [147]. It can be of two types: essential and symptomatic. Symptomatic or secondary cases are associated with structural brain lesions ranging from demyelinating to space-occupying lesions involving GMT. Ear clicks are an important clinical correlate seen in essential palatal myoclonus due to the involvement of the tensor vali palatini muscle, which helps differentiate it from symptomatic palatal myoclonus where there are no ear clicks [148]. The lesions in the palatal myoclonus are thought to be localized to the dentato-rubro-olivary pathway [147,185]. Other types of myoclonus reported in patients with MS are intention myoclonus [178,182,187], middle ear myoclonus [183], and spinal myoclonus [180,181]. It is controversial whether middle ear myoclonus is different or is a part of palatal myoclonus. Middle ear myoclonus is associated with tinnitus and ear clicks [183]. Tensor tympani contractions, two walls of Eustachian tube collision, and stapedius contractions are proposed mechanisms underlying middle ear myoclonus [183].

Intention myoclonus is rarely seen in MS [178] and has been related to neuronal loss contributed by demyelination in the red nuclei [178]. Myoclonic jerks may resemble flexor spasms, a frequent finding in patients with spasticity and MS. Hence, a careful clinical distinction between the two may aid in correct clinical interpretation and treatment.

Spinal segmental myoclonus, characterized by involuntary, semirhythmic contractions of skeletal muscle groups innervated by a limited spinal cord region, poses a diagnostic challenge at the time of presentation. It is usually precipitated by fatigue, stress, and relieved in sleep. Among six cases of demyelinating disorder associated with myoclonus, Jankovic and Pardo [186] reported one with spinal myoclonus and five others with brainstem myoclonus. Due to demyelinating lesions at the cervical roots, upper limb myoclonus has been reported [180,181,184]. The possible pathophysiological mechanisms include axonal hyperexcitability and spontaneous discharge, leading to the disinhibition of alpha-motor neurons and disrupted spinal interneuronal circuitry [180,188].

Commonly used therapeutics that can be effective are valproic acid, clonazepam, tizanidine, and levetiracetam [121,182,187]. Botulinum toxin could prove beneficial in palatal myoclonus in MS [189]. Medical management was unsuccessful in an MS case of bilateral middle ear myoclonus causing incapacitating tinnitus [183], which was successfully treated with bilateral sectioning of tensor tympani and stapedial tendons.

### 6) Ballism

Several reports of hemiballismus in patients with MS have been reported [147,190,191,192,193,194]. In two reports, MS defining demyelinating plaques were observed in the contralateral subthalamic nucleus [191,194]. The treatment options for ballism are almost the same as chorea, except it may be reasonable to start with corticosteroids. Neuroleptics may also be used. However, neuroleptics should be used with caution in patients with MS in general, as they have been reported to cause, albeit rarely, adverse reactions [195].

### 7) Facial Myokymia

Facial myokymia is frequently reported as the presenting feature in patients with MS [196,197,198,199,200,201,202,203,204,205,206,207,208,209,210,211]. The lesion is usually attributed to the postnuclear facial fascicular involvement in the dorsolateral pontine tegmentum [212]. Strictly unilateral myokymia involving peri-oral muscles warrants a search for underlying structural/demyelinating lesion over pons in contrast to eyelid myokymia, which is usually benign without any structural correlations [206].

Facial myokymia is often resolved spontaneously. A descriptive study showed that most facial myokymia in MS remits regardless of treatment received [208]. When it does not resolve, it may progress into a lower motor neuron type facial palsy [97,209]. Thus, a progressive or persistent myokymia (more than six months) should also raise the suspicion of secondary causes such as MS. Most patients respond to corticosteroids, gabapentin, carbamazepine, and botulinum toxin [52,202,208].

### 8) Hemifacial Spasm

A prospective observational study in a cohort of 60 patients with MS revealed that 58.3% had demyelination-related movement disorders. Two of them were found to have hemifacial spasms secondary to pontine demyelination [1]. In a descriptive study of clinical features and treatment outcomes involving 35 patients with MS, seven had hemifacial spasm [208]. In another case series of six patients with MS who developed hemifacial spasm, two had unilateral lower pontine lesions visible in brain MRI [197]. The involvement of the platysma is characteristic of the idiopathic variety, whereas in secondary causes, the upper and lower facial muscles are simultaneously involved [97,213].

Spastic paretic hemifacial contracture, associated initially with brainstem tumors, has also been described in a few cases of MS [211,214,215,216]. Koutsis et al [214]. screened 500 patients with MS and found two cases of spastic paretic hemifacial contracture, which were characterized by continuous resting activity by irregular motor unit firing potentials on electromyogram and the absence of myokymic discharges. The lesions have been attributed to the involvement of ipsilateral dorsolateral pontine tegmentum [211,216] and demyelinated corticofacial fibers [216].

Several theories exist regarding the pathophysiology of hemifacial spasms in MS. Demyelination can cause ephaptic transmission, leading to abnormal firing [217]. The cranial nerves’ transition zones (i.e., root-exit zones) are susceptible to injury. Irritative feedback from peripheral lesions can also cause hyperexcitability of the facial nerve nucleus. Consequently, microvascular decompression (for facial palsy and trigeminal neuralgia) and radiofrequency rhizotomy (trigeminal neuralgia) could benefit [208]. Most hemifacial spasm cases in MS resolve (almost 71%), regardless of treatment [208].

### 9) Tics and tourettism

The coexistence of MS with tics and tourettism is extremely rare, with two reports of tourettism [218], one report of complex vocal tic [219], and another one with simple phonic tics [220]. In Nociti et al. [218], the tourettism was presumed secondary to progressive MS. The anatomical localization of tics has not been established. Involvement of the cortico-striatal-thalamocortical circuit and basal ganglia is supported by clinical evidence, as demyelinating lesions were observed disrupting the basal ganglia and thalamus circuits [218,219,220].

The patient with secondary progressive MS and tourettism was successfully treated with quetiapine [218]. The one with simple phonic tics presented with paroxysmal throat-clearing sounds recovered with pimozide [220]. Available treatment options include typical neuroleptics (e.g., haloperidol, pimozide), α-adrenergic receptor agonists (e.g., clonidine), and atypical neuroleptics (e.g., clozapine, risperidone), tetrabenazine, and carbamazepine, among others [121,221]. Finally, DBS may be helpful as a third-line treatment in patients who are refractory to medical treatment [222].

### 10) Parkinsonism

Parkinsonism is a rare phenomenon in MS [147,172,223,224,225,226,227,228,229,230,231,232,233,234,235,236,237,238,239,240,241,242,243]. The association may be coincidental [147]. or caused by MS [226,231]. Evidence supporting a causal relationship includes the lesions of basal ganglia or midbrain on neuroimaging and improvement with corticosteroids [225,226,231,240,243]. Demyelinating plaques involving the basal ganglia and thalamus are quite common in patients with MS [244], and involvement of nigrostriatal pathway may lead to features of parkinsonism [244,245]. The reports with either documented evidence of lesions on neuroimaging (i.e., MRI) or therapeutic response to corticosteroids are summarized in ***Table 3***.

**Table 3:** Reported cases having parkinsonism in multiple sclerosis with either brain MRI lesions related to Parkinson’s disease or a positive response to corticosteroids.


AUTHOR, YEAR	CASES (AGE, SEX)	MRI LESIONS IN MS-RELATED TO PARKINSONISM	L-DOPA RESPONSE	CORTICOSTEROID RESPONSE

Vieregge et al. 1992 [225]	2 cases (55/M; 60/F)	M: periventricular white matter, left lateral thalamus, globus pallidus F: periventricular white matter, lateral thalamus	–	M: Yes F: Yes

Federlein et al. 1997 [226]	1 case (61/M)	Substantia nigra	–	Yes

Folgar et al. 2003 [231]	1 case (48/F)	Periventricular white matter	–	Yes

Wittstock et al. 2001 [243]	1 case (58/F)	Periventricular region, substantia nigra	–	Yes

Burn and Cartlidge 1996 [240]	1 case (45/F)	Paraventricular areas	–	Yes

Tranchant et al. 1995 [147]	1 case (46/F)	Cerebral peduncle near substantia nigra	–	–

Maranhao et al. 1995 [237]	1 case (48/M)	Cerebral peduncles, thalamus, globus pallidus	–	–

Ozturk et al. 2002 [228]	1 case (39/F)	Substantia nigra	Yes	–

Barun et al. 2008 [227]	2 cases (38/F; 53/F)	1^st^ F: Basal ganglia 2^nd^ F: periventricular white matter	Yes	Yes

Valkovic et al. 2007 [223]	1 case (25/M)	Subthalamic region	Yes	–

Kreisler et al. 2004 [239]	1 case (38/F)	Substantia nigra	Yes	–

Saidha et al. 2010 [232]	1 case (53/M)	Substantia nigra, thalamus, globus pallidus	Yes	Yes

Schultheiss et al. 2011 [230]	1 case (82/M)	Basal ganglia, periventricular white matter	–	–

Etemadifar et al. 2014 [236]	8 cases (5F: 32.6 ± 7; 3M: 34.6 ± 6.8)	Basal ganglia (four cases), thalamus (two cases), midbrain (five cases)	Yes (all)	–

Bougea et al. 2015 [235]	1 case (55/F)	Periventricular white matter, thalamus	Yes	–

Delalic et al. 2020 [234]	1 case (52/F)	White matter	Yes	Yes (initially)

Shaygannejad et al. 2016 [233]	1 case (21/F)	Periventricular	Yes	Yes (initially)


The age is mentioned according to the onset of multiple sclerosis. F = Female; M = Male. MRI = magnetic resonance imaging.

The frequency of basal ganglia lesions on MRI of patients with MS and the rarity of parkinsonism in the setting of MS and normal MRI in the presence of parkinsonian features also suggests that these lesions may not be causal [223,239]. Any such correlating features are absent in coincidental association, and those cases are responsive to levodopa [223,224,227]. Indeed, a nationwide historical prospective study on a Danish cohort did not find any increased risk of Parkinson’s disease in MS (standardized incidence ratio 0.98, 95% CI 0.67-1.44), which suggests the absence of a causal association [246].

However, recent genetic evidence may indicate a possible relationship between the two disorders. The increased expression of α-synuclein has been observed in astrocytes in normal-appearing white matter adjacent to MS lesions in secondary progressive MS [247]. Neuronal loss was observed in both white matter and grey matter structures (e.g., thalamus), suggesting that immune-mediated demyelinating diseases may share some standard features with other neurodegenerative conditions such as parkinsonism. In addition, increased cerebrospinal fluid (CSF) α-synuclein in patients with MS may suggest axonal injury around inflammatory lesions [248]. PARK2 gene, associated with young-onset parkinsonism, is highly expressed in acute plaques in patients with MS [205]. Similarly, PTEN-induced kinase 1, which has a protective role against stress-induced mitochondrial dysfunction, showed marked astrocytic immunostaining in demyelinating lesions of MS [249]. The genetic variability of HLA-DRB5 is also evidence in favor of a possible genetic relationship between MS and parkinsonism as it has a role in the inflammatory processes in both diseases [250].

## Conclusion

MS is a debilitating disease with severe implications for its sufferers’ health-related quality of life. Movement disorders are relatively common in MS but have varied manifestations, underlying pathomechanisms, and treatment modalities. In many instances, movement disorders are presenting features, indicating the importance of these pathologies in the early diagnosis of MS. The most prevalent movement disorders in MS include RLS, tremor, ataxia, parkinsonism, paroxysmal dyskinesias, dystonia, chorea and ballism, facial myokymia, including hemifacial spasm and spastic paretic hemifacial contracture, tics, and tourettism.

A multidisciplinary approach including pharmacologic, surgical, and physical therapy is usually necessary for MS-associated tremor and ataxia. Newer methods such as focused ultrasound thalamotomy may be appropriate for MS-tremor management because they are cost-effective, time-saving, minimally invasive, and have reduced infection risk, however, further studies are needed. Treatment options are based on previous case studies and expert opinions rather than high-quality clinical and epidemiological evidence. Advancement of our understanding of movement disorders in MS regarding their prevalence, impact on the patient population, and management options are only possible when these issues are addressed in large cohorts. Until then, awareness among clinicians may aid in avoiding delays in the management of these patients.

## Additional File

The additional file for this article can be found as follows:

10.5334/tohm.671.s1 Supplementary Table 1. Search terms used on PubMed platform, October 2021.
