# Supplementary material for: Movement Disorders in Multiple Sclerosis: An Update
Source: Tremor Other Hyperkinet Mov (N Y). 2022 May 4;12:14. doi: 10.5334/tohm.671 (PMC9075048; doi:10.5334/tohm.671)
Supplement: Supplementary Table 1. — Search terms used on PubMed platform, October 2021. [file tohm-12-1-671-s1.pdf]

Supplementary Table 1: Search terms used on PubMed platform, October 2021.

No.	Search terms	Total results
1.	Tremor AND Multiple Sclerosis	619
2.	Restless leg syndrome AND Multiple sclerosis	120
3.	Ataxia AND Multiple Sclerosis	902
4.	Paroxysmal dyskinesia AND Multiple Sclerosis	322
5.	Dystonia AND Multiple Sclerosis	261
6.	Chorea AND Multiple Sclerosis	311
7.	Ballism AND Multiple Sclerosis	1012
8.	Parkinsonism AND Multiple Sclerosis	3815
9.	Tics AND Multiple Sclerosis	15
10.	Tourettism AND Multiple Sclerosis	70
11.	Myokymia AND Multiple Sclerosis	49
12.	Hemifacial spasms AND Multiple Sclerosis	21
13.	Spastic paretic hemifacial contracture AND Multiple Sclerosis	4
14.	4 OR 5 OR 6 OR 7	1188
15.	9 OR 10	79
16.	11 OR 12 OR 13	65
17.	1 OR 2 OR 3 OR 8 OR 14 OR 15 OR 16	5334
